# Supplementary material for: Sulfonylureas may be useful for glycemic management in patients with diabetes and liver cirrhosis
Source: PLoS One. 2020 Dec 14;15(12):e0243783. doi: 10.1371/journal.pone.0243783 (PMC7735585; doi:10.1371/journal.pone.0243783)
Supplement: S1 Table — (DOCX) [file pone.0243783.s001.docx]

**S1 Table. Stratified analysis of all-cause mortality associated with sulfonylurea use and nonuse.**

| **Variable** | **Non-sulfonylurea users**  **(n= 3781)** | | | **Sulfonylurea users**  **(n= 3781)** | | | **Crude HR (95% CI)** | **Adjusted HR  (95% CI) ^a^** | **p for**  **interaction** |
| --- | --- | --- | --- | --- | --- | --- | --- | --- | --- |
|  | **Events** | **PY** | **IR** | **Events** | **PY** | **IR** |  |  |  |
| Overall | 918 | 22420 | 4.09 | 680 | 20984 | 3.24 | 0.83(0.75-0.92)*** | 0.79(0.71-0.88)*** |  |
| Glibenclamide | 918 | 22420 | 4.09 | 324 | 9097 | 3.56 | 0.85(0.75-0.97)* | 0.84(0.74-0.95)** |  |
| Glipizide/ Gliclazide | 918 | 22420 | 4.09 | 269 | 8294 | 3.24 | 0.81(0.71-0.93)** | 0.77(0.67-0.88)*** |  |
| Glimepiride | 918 | 22420 | 4.09 | 75 | 3068 | 2.44 | 0.80(0.63-0.98)* | 0.67(0.52-0.86)** |  |
| Age group, years |  |  |  |  |  |  |  |  | 0.55 |
| 18-49 | 235 | 6577 | 3.57 | 150 | 5899 | 2.54 | 0.73(0.60-0.90)** | 0.71(0.58-0.88)** |  |
| 50-65 | 418 | 10883 | 3.84 | 314 | 10253 | 3.06 | 0.84(0.73-0.98)* | 0.78(0.67-0.91)** |  |
| >65 | 265 | 4959 | 5.34 | 216 | 4831 | 4.47 | 0.87(0.72-1.04) | 0.89(0.75-1.07) |  |
| Sex |  |  |  |  |  |  |  |  | 0.13 |
| Female | 255 | 7592 | 3.36 | 211 | 7090 | 2.98 | 0.94(0.78-1.13) | 0.89(0.74-1.08) |  |
| Male | 663 | 14827 | 4.47 | 469 | 13893 | 3.38 | 0.79(0.70-0.88)*** | 0.76(0.68-0.86)*** |  |
| Antihypertensive drugs |  |  |  |  |  |  |  |  |  |
| ACEI/ARB |  |  |  |  |  |  |  |  | 0.81 |
| No | 493 | 12198 | 4.04 | 359 | 11293 | 3.18 | 0.84(0.73-0.96)* | 0.83(0.72-0.95)** |  |
| Yes | 425 | 10221 | 4.16 | 321 | 9691 | 3.31 | 0.82(0.70-0.94)** | 0.85(0.73-0.98)* |  |
| β-blockers |  |  |  |  |  |  |  |  | 0.69 |
| No | 410 | 9866 | 4.16 | 289 | 9072 | 3.19 | 0.81(0.69-0.94)** | 0.82(0.71-0.96)* |  |
| Yes | 508 | 12553 | 4.05 | 391 | 11912 | 3.28 | 0.83(0.72-0.95)* | 0.84(0.74-0.96)* |  |
| Calcium-channel blockers |  |  |  |  |  |  |  |  | 0.76 |
| No | 636 | 15495 | 4.10 | 455 | 14297 | 3.18 | 0.82(0.73-0.92)** | 0.82(0.72-0.92)** |  |
| Yes | 282 | 6924 | 4.07 | 225 | 6687 | 3.36 | 0.84(0.70-0.99)* | 0.86(0.72-1.03) |  |
| Diuretics |  |  |  |  |  |  |  |  | 0.42 |
| No | 676 | 16288 | 4.15 | 502 | 15073 | 3.33 | 0.85(0.76-0.96)** | 0.84(0.75-0.94)** |  |
| Yes | 242 | 6131 | 3.95 | 178 | 5911 | 3.01 | 0.77(0.64-0.94)** | 0.76(0.62-0.92)** |  |
| Antidiabetic drugs |  |  |  |  |  |  |  |  |  |
| Metformin |  |  |  |  |  |  |  |  | 0.04 |
| No | 539 | 13335 | 4.04 | 433 | 12594 | 3.44 | 0.85(0.76-0.94)** | 0.88(0.78-0.99)* |  |
| Yes | 379 | 9084 | 4.17 | 247 | 8390 | 2.94 | 0.68(0.50-0.90)** | 0.75(0.64-0.89)*** |  |
| Meglitinide |  |  |  |  |  |  |  |  | 0.16 |
| No | 812 | 20086 | 4.04 | 603 | 18556 | 3.25 | 0.86(0.77-0.94)** | 0.84(0.76-0.93)** |  |
| Yes | 106 | 2333 | 4.54 | 77 | 2428 | 3.17 | 0.68(0.51-0.92)** | 0.62(0.46-0.84)** |  |
| Thiazolidinedione |  |  |  |  |  |  |  |  | 0.77 |
| No | 801 | 19760 | 4.05 | 578 | 18359 | 3.15 | 0.82(0.74-0.91)*** | 0.84(0.75-0.93)*** |  |
| Yes | 117 | 2659 | 4.40 | 102 | 2625 | 3.89 | 0.85(0.65-1.11) | 0.90(0.68-1.18) |  |
| α-glucosidase inhibitor |  |  |  |  |  |  |  |  | 0.32 |
| No | 820 | 20148 | 4.07 | 605 | 18646 | 3.24 | 0.84(0.76-0.93)** | 0.82(0.73-0.91)** |  |
| Yes | 98 | 2271 | 4.32 | 75 | 2338 | 3.21 | 0.71(0.53-0.97)* | 0.73(0.54-1.00) |  |
| DPP-4 inhibitors |  |  |  |  |  |  |  |  | 0.14 |
| No | 904 | 22021 | 4.11 | 675 | 20612 | 3.27 | 0.83(0.76-0.92)*** | 0.83(0.75-0.92)*** |  |
| Yes | 14 | 399 | 3.51 | 5 | 372 | 1.34 | 0.39(0.14-1.07) | 0.44(0.13-1.47) |  |
| Insulin |  |  |  |  |  |  |  |  | 0.60 |
| No | 724 | 17800 | 4.07 | 535 | 16541 | 3.23 | 0.83(0.75-0.93)** | 0.83(0.75-0.93)** |  |
| Yes | 194 | 4619 | 4.20 | 145 | 4443 | 3.26 | 0.78(0.63-0.97)* | 0.79(0.63-0.98)* |  |
| Other drugs |  |  |  |  |  |  |  |  |  |
| Statin |  |  |  |  |  |  |  |  | 0.64 |
| No | 673 | 16488 | 4.08 | 498 | 15394 | 3.24 | 0.84(0.75-0.94)** | 0.84(0.75-0.94)** |  |
| Yes | 245 | 5932 | 4.13 | 182 | 5590 | 3.26 | 0.76(0.63-0.92)** | 0.80(0.66-0.97)** |  |
| Aspirin |  |  |  |  |  |  |  |  | 0.89 |
| No | 340 | 8616 | 3.95 | 241 | 7934 | 3.04 | 0.82(0.69-0.97)* | 0.80(0.68-0.95)* |  |
| Yes | 578 | 13803 | 4.19 | 439 | 13050 | 3.36 | 0.83(0.73-0.94)** | 0.84(0.74-0.95)** |  |
| DCSI score |  |  |  |  |  |  |  |  | 0.20 |
| 0 | 413 | 9487 | 4.35 | 277 | 9031 | 3.07 | 0.74(0.64-0.87)*** | 0.73(0.63-0.86)*** |  |
| 1 | 174 | 4574 | 3.80 | 138 | 4221 | 3.27 | 0.90(0.72-1.13) | 0.88(0.70-1.11) |  |
| ≥2 | 331 | 8357 | 3.96 | 265 | 7732 | 3.43 | 0.89(0.76-1.05) | 0.91(0.77-1.07) |  |
| CCI index |  |  |  |  |  |  |  |  | 0.48 |
| 0 | 540 | 13764 | 3.92 | 403 | 12907 | 3.12 | 0.84(0.74-0.96)** | 0.84(0.73-0.95)** |  |
| 1 | 170 | 3726 | 4.56 | 106 | 3351 | 3.16 | 0.72(0.57-0.92)** | 0.69(0.55-0.89)** |  |
| ≥2 | 208 | 4929 | 4.22 | 171 | 4725 | 3.62 | 0.88(0.72-1.08) | 0.90(0.73-1.11) |  |
| Smoking |  |  |  |  |  |  |  |  | 0.32 |
| No | 881 | 21189 | 4.16 | 646 | 19824 | 3.26 | 0.82(0.74-0.91)*** | 0.81(0.74-0.90)*** |  |
| Yes | 37 | 1230 | 3.01 | 34 | 1160 | 2.93 | 1.01(0.63-1.61) | 1.08(0.66-1.75) |  |
| Comorbidity |  |  |  |  |  |  |  |  |  |
| Hypertension |  |  |  |  |  |  |  |  | 0.77 |
| No | 563 | 13259 | 4.25 | 415 | 12499 | 3.32 | 0.82(0.72-0.93)** | 0.81(0.71-0.92)** |  |
| Yes | 355 | 9160 | 3.88 | 265 | 8485 | 3.12 | 0.84(0.72-0.98)* | 0.85(0.73-1.00) |  |
| Dyslipidemia |  |  |  |  |  |  |  |  | 0.86 |
| No | 604 | 14345 | 4.21 | 448 | 13436 | 3.33 | 0.83(0.73-0.94)** | 0.82(0.73-0.93)** |  |
| Yes | 314 | 8075 | 3.89 | 232 | 7548 | 3.07 | 0.81(0.69-0.96)* | 0.83(0.69-0.98)* |  |
| CKD |  |  |  |  |  |  |  |  | 0.64 |
| No | 773 | 19000 | 4.07 | 576 | 17772 | 3.24 | 0.83(0.74-0.93)** | 0.83(0.74-0.92)*** |  |
| Yes | 145 | 3419 | 4.24 | 104 | 3211 | 3.24 | 0.78(0.61-1.01) | 0.78(0.61-1.01) |  |
| COPD |  |  |  |  |  |  |  |  | 0.43 |
| No | 753 | 18363 | 4.10 | 559 | 17068 | 3.28 | 0.84(0.76-0.94)** | 0.83(0.74-0.93)** |  |
| Yes | 165 | 4056 | 4.07 | 121 | 3916 | 3.09 | 0.76(0.61-0.97)* | 0.78(0.61-0.99)* |  |
| HBV |  |  |  |  |  |  |  |  | 0.51 |
| No | 757 | 18603 | 4.07 | 531 | 16986 | 3.13 | 0.81(0.72-0.90)*** | 0.81(0.72-0.90)*** |  |
| Yes | 161 | 3816 | 4.22 | 149 | 3998 | 3.73 | 0.88(0.71-1.10) | 0.90(0.72-1.14) |  |
| HCV |  |  |  |  |  |  |  |  | 0.89 |
| No | 791 | 19396 | 4.08 | 572 | 17933 | 3.19 | 0.82(0.73-0.91)*** | 0.81(0.73-0.91)*** |  |
| Yes | 127 | 3024 | 4.20 | 108 | 3051 | 3.54 | 0.84(0.65-1.09) | 0.87(0.67-1.14) |  |

^*^*p* < 0.05, ^**^*p* < 0.01, ^***^*p* < 0.001. PY, person-years; IR, incidence rate, per 100 person-years; HR, hazard ratio; CI, confidence interval; ACEI, angiotensin converting enzyme inhibitor; ARB, angiotensin receptor blocker; CCI, Charlson comorbidity index; DCSI score, diabetes complications severity index score; HBV, hepatitis B virus; DM, diabetes mellitus; HCV, hepatitis C virus. **^a^**Adjusted for age, sex, index year, age at DM diagnosis, DM duration (years), antihypertensive drugs (ACE inhibitors, ARBs, β-blockers, calcium-channel blockers, diuretics, other antihypertensive), antidiabetic drugs (metformin, meglitinides, thiazolidinedione, α-glucosidase inhibitor, DPP-4 inhibitors, insulin), statin, aspirin, CCI index (0, 1, ≥2), DCSI score (0, 1, ≥2), obesity, smoking, hypertension, dyslipidemia, CKD, COPD, HBV, and HCV.
