# Supplementary material for: Sulfonylureas may be useful for glycemic management in patients with diabetes and liver cirrhosis
Source: PLoS One. 2020 Dec 14;15(12):e0243783. doi: 10.1371/journal.pone.0243783 (PMC7735585; doi:10.1371/journal.pone.0243783)
Supplement: S2 Table — (DOCX) [file pone.0243783.s002.docx]

**S2 Table. Stratified analysis of major adverse cardiovascular events associated with sulfonylurea use and nonuse.**

| **Variable** | **Non-sulfonylurea users**  **(n= 3781)** | | | **Sulfonylurea users**  **(n= 3781)** | | | **Crude HR (95% CI)** | **Adjusted HR  (95% CI) ^a^** | **p for**  **interaction** |
| --- | --- | --- | --- | --- | --- | --- | --- | --- | --- |
|  | **Events** | **PY** | **IR** | **Events** | **PY** | **IR** |  |  |  |
| Overall | 559 | 20424 | 2.74 | 363 | 19946 | 1.82 | 0.66(0.58-0.76)*** | 0.69(0.61-0.80)*** |  |
| Glibenclamide | 559 | 20424 | 2.74 | 172 | 8612 | 2.00 | 0.72(0.61-0.86)*** | 0.73(0.61-0.87)*** |  |
| Glipizide/ Gliclazide | 559 | 20424 | 2.74 | 151 | 7837 | 1.93 | 0.70(0.59-0.84)*** | 0.71(0.59-0.85)*** |  |
| Glimepiride | 559 | 20424 | 2.74 | 35 | 2978 | 1.18 | 0.44(0.31-0.62)*** | 0.55(0.38-0.77)*** |  |
| Age group, years |  |  |  |  |  |  |  |  | 0.10 |
| 18-49 | 135 | 6127 | 2.20 | 67 | 5721 | 1.17 | 0.54(0.40-0.72)*** | 0.51(0.38-0.69)*** |  |
| 50-65 | 263 | 9890 | 2.66 | 167 | 9784 | 1.71 | 0.64(0.52-0.78)*** | 0.67(0.55-0.82)*** |  |
| >65 | 161 | 4406 | 3.65 | 129 | 4440 | 2.91 | 0.79(0.63-1.00) | 0.85(0.67-1.08) |  |
| Sex |  |  |  |  |  |  |  |  | 0.36 |
| Female | 180 | 6896 | 2.61 | 127 | 6718 | 1.89 | 0.73(0.57-0.91)** | 0.78(0.62-0.99)* |  |
| Male | 379 | 13528 | 2.80 | 236 | 13228 | 1.78 | 0.63(0.54-0.75)*** | 0.66(0.56-0.78)*** |  |
| Antihypertensive drugs |  |  |  |  |  |  |  |  |  |
| ACEI/ARB |  |  |  |  |  |  |  |  | 0.62 |
| No | 292 | 11063 | 2.64 | 183 | 10778 | 1.70 | 0.64(0.53-0.77)*** | 0.64(0.54-0.77)*** |  |
| Yes | 267 | 9361 | 2.85 | 180 | 9168 | 1.96 | 0.69(0.57-0.83)*** | 0.71(0.58-0.86)*** |  |
| β-blockers |  |  |  |  |  |  |  |  | 0.23 |
| No | 231 | 9023 | 2.56 | 129 | 8666 | 1.49 | 0.58(0.47-0.72)*** | 0.63(0.51-0.78)*** |  |
| Yes | 328 | 11401 | 2.88 | 234 | 11280 | 2.07 | 0.72(0.61-0.85)*** | 0.72(0.60-0.85)*** |  |
| Calcium-channel blockers |  |  |  |  |  |  |  |  | 0.26 |
| No | 399 | 13971 | 2.86 | 245 | 13574 | 1.80 | 0.63(0.54-0.74)*** | 0.62(0.53-0.73)*** |  |
| Yes | 160 | 6453 | 2.48 | 118 | 6372 | 1.85 | 0.75(0.59-0.95)* | 0.78(0.61-0.99)* |  |
| Diuretics |  |  |  |  |  |  |  |  | 0.06 |
| No | 423 | 14730 | 2.87 | 253 | 14336 | 1.76 | 0.62(0.53-0.72)*** | 0.60(0.52-0.71)*** |  |
| Yes | 136 | 5694 | 2.39 | 110 | 5610 | 1.96 | 0.82(0.63-1.05) | 0.81(0.63-1.05) |  |
| Antidiabetic drugs |  |  |  |  |  |  |  |  |  |
| Metformin |  |  |  |  |  |  |  |  | 0.08 |
| No | 350 | 11981 | 2.92 | 251 | 11811 | 2.13 | 0.73(0.62-0.86)*** | 0.72(0.61-0.85)*** |  |
| Yes | 209 | 8443 | 2.48 | 112 | 8135 | 1.38 | 0.55(0.44-0.69)*** | 0.58(0.46-0.74)*** |  |
| Meglitinide |  |  |  |  |  |  |  |  | 0.008 |
| No | 520 | 18174 | 2.86 | 317 | 17637 | 1.80 | 0.63(0.54-0.72)*** | 0.62(0.54-0.71)**** |  |
| Yes | 39 | 2250 | 1.73 | 46 | 2308 | 1.99 | 1.14(0.74-1.75) | 1.15(0.74-1.78) |  |
| Thiazolidinedione |  |  |  |  |  |  |  |  | 0.29 |
| No | 502 | 17896 | 2.81 | 317 | 17429 | 1.82 | 0.65(0.56-0.75)*** | 0.64(0.56-0.74)*** |  |
| Yes | 57 | 2528 | 2.25 | 46 | 2517 | 1.83 | 0.79(0.53-1.17) | 0.78(0.52-1.18) |  |
| α-glucosidase inhibitor |  |  |  |  |  |  |  |  | 0.85 |
| No | 516 | 18229 | 2.83 | 332 | 17670 | 1.88 | 0.66(0.58-0.76)*** | 0.66(0.57-0.76)*** |  |
| Yes | 43 | 2195 | 1.96 | 31 | 2276 | 1.36 | 0.69(0.44-1.10) | 0.68(0.42-1.11) |  |
| DPP-4 inhibitors |  |  |  |  |  |  |  |  | 0.23 |
| No | 553 | 20030 | 2.76 | 362 | 19575 | 1.85 | 0.67(0.59-0.77)*** | 0.66(0.58-0.76)*** |  |
| Yes | 6 | 394 | 1.52 | 1 | 370 | 0.27 | 0.18(0.02-1.48) | 0.32(0.01-1.10) |  |
| Insulin |  |  |  |  |  |  |  |  | 0.81 |
| No | 458 | 16059 | 2.85 | 295 | 15669 | 1.88 | 0.66(0.57-0.76)*** | 0.65(0.57-0.76)*** |  |
| Yes | 101 | 4365 | 2.31 | 68 | 4277 | 1.59 | 0.69(0.51-0.94)* | 0.68(0.50-0.93)* |  |
| Other drugs |  |  |  |  |  |  |  |  |  |
| Statin |  |  |  |  |  |  |  |  | 0.66 |
| No | 411 | 14889 | 2.76 | 272 | 14583 | 1.87 | 0.68(0.58-0.79)*** | 0.67(0.57-0.77)*** |  |
| Yes | 148 | 5535 | 2.67 | 91 | 5363 | 1.70 | 0.63(0.49-0.82)** | 0.62(0.48-0.81)** |  |
| Aspirin |  |  |  |  |  |  |  |  | 0.43 |
| No | 203 | 7848 | 2.59 | 138 | 7501 | 1.84 | 0.72(0.58-0.89)** | 0.75(0.60-0.93)** |  |
| Yes | 356 | 12576 | 2.83 | 225 | 12445 | 1.81 | 0.64(0.54-0.75)*** | 0.63(0.53-0.75)*** |  |
| DCSI score |  |  |  |  |  |  |  |  | 0.61 |
| 0 | 256 | 8500 | 3.01 | 159 | 8549 | 1.86 | 0.62(0.51-0.75)*** | 0.62(0.51-0.76)*** |  |
| 1 | 104 | 4223 | 2.46 | 68 | 4003 | 1.70 | 0.69(0.51-0.94)* | 0.66(0.48-0.91)** |  |
| ≥2 | 199 | 7701 | 2.58 | 136 | 7393 | 1.84 | 0.72(0.58-0.89)** | 0.71(0.57-0.88)** |  |
| CCI index |  |  |  |  |  |  |  |  | 0.42 |
| 0 | 277 | 12771 | 2.17 | 188 | 12372 | 1.52 | 0.70(0.59-0.85)*** | 0.70(0.58-0.85)*** |  |
| 1 | 133 | 3212 | 4.14 | 73 | 3125 | 2.34 | 0.56(0.42-0.75)*** | 0.55(0.41-0.73)*** |  |
| ≥2 | 149 | 4440 | 3.36 | 102 | 4448 | 2.29 | 0.68(0.53-0.88)** | 0.67(0.52-0.86)** |  |
| Smoking |  |  |  |  |  |  |  |  | 0.16 |
| No | 533 | 19303 | 2.76 | 338 | 18845 | 1.79 | 0.65(0.57-0.74)*** | 0.64(0.56-0.73)*** |  |
| Yes | 26 | 1121 | 2.32 | 25 | 1101 | 2.27 | 0.96(0.55-1.67) | 0.84(0.45-1.50) |  |
| Comorbidity |  |  |  |  |  |  |  |  |  |
| Hypertension |  |  |  |  |  |  |  |  | 0.08 |
| No | 299 | 12212 | 2.45 | 215 | 11888 | 1.81 | 0.74(0.62-0.88)*** | 0.73(0.61-0.87)*** |  |
| Yes | 260 | 8212 | 3.17 | 148 | 8058 | 1.84 | 0.57(0.47-0.71)*** | 0.58(0.48-0.71)*** |  |
| Dyslipidemia |  |  |  |  |  |  |  |  | 0.36 |
| No | 326 | 13179 | 2.47 | 222 | 12834 | 1.73 | 0.70(0.59-0.83)*** | 0.69(0.58-0.82)*** |  |
| Yes | 233 | 7245 | 3.22 | 141 | 7112 | 1.98 | 0.62(0.50-0.76)*** | 0.61(0.49-0.75)*** |  |
| CKD |  |  |  |  |  |  |  |  | 0.75 |
| No | 455 | 17408 | 2.61 | 291 | 16949 | 1.72 | 0.65(0.57-0.76)*** | 0.65(0.56-0.75)*** |  |
| Yes | 104 | 3016 | 3.45 | 72 | 2997 | 2.40 | 0.69(0.51-0.93)* | 0.70(0.52-0.95)* |  |
| COPD |  |  |  |  |  |  |  |  | 0.79 |
| No | 439 | 16839 | 2.61 | 284 | 16280 | 1.74 | 0.67(0.57-0.78)*** | 0.67(0.58-0.78)** |  |
| Yes | 120 | 3585 | 3.35 | 79 | 3666 | 2.15 | 0.64(0.48-0.86)** | 0.64(0.48-0.85)** |  |
| HBV |  |  |  |  |  |  |  |  | 0.43 |
| No | 467 | 16904 | 2.76 | 289 | 16135 | 1.79 | 0.65(0.56-0.75)*** | 0.64(0.55-0.74)*** |  |
| Yes | 92 | 3520 | 2.61 | 74 | 3811 | 1.94 | 0.75(0.55-1.02) | 0.73(0.53-1.00) |  |
| HCV |  |  |  |  |  |  |  |  | 0.66 |
| No | 499 | 17579 | 2.84 | 318 | 16998 | 1.87 | 0.66(0.57-0.76)*** | 0.65(0.57-0.75)*** |  |
| Yes | 60 | 2845 | 2.11 | 45 | 2948 | 1.53 | 0.73(0.49-1.07) | 0.73(0.49-1.09) |  |

^*^*p* < 0.05, ^**^*p* < 0.01, ^***^*p* < 0.001. PY, person-years; IR, incidence rate, per 100 person-years; HR, hazard ratio; CI, confidence interval; ACEI, angiotensin converting enzyme inhibitor; ARB, angiotensin receptor blocker; CCI, Charlson comorbidity index; DCSI score, diabetes complications severity index score; HBV, hepatitis B virus; DM, diabetes mellitus; HCV, hepatitis C virus. ^a^Adjusted for age, sex, index year, age at DM diagnosis, DM duration (years), antihypertensive drugs (ACE inhibitors, ARBs, β-blockers, calcium-channel blockers, diuretics, other antihypertensive), antidiabetic drugs (metformin, meglitinides, thiazolidinedione, α-glucosidase inhibitor, DPP-4 inhibitors, insulin), statin, aspirin, CCI index (0, 1, ≥2), DCSI score (0, 1, ≥2), obesity, smoking, hypertension, dyslipidemia, CKD, COPD, HBV, and HCV.
